# Can Shared Decision Making Improve Physician Well-Being and Reduce Burnout?

**DOI:** 10.7759/cureus.1615

**Published:** 2017-08-27

**Authors:** Claudia C Dobler, Colin P West, Victor M Montori

**Affiliations:** 1 Knowledge and Evaluation Research Unit, Mayo Clinic, Rochester, Minnesota; 2 Division of General Internal Medicine and Division of Biomedical Statistics and Informatics, Mayo Clinic, Rochester, Minnesota

**Keywords:** shared deicion making, physician well-being, burnout, clinical encounter, interaction

## Abstract

There are many causes of physician burnout in today’s health care environment, including an ever increasing administrative workload, pressure to do more work in less time, and a drive to reduce costs and improve patient outcomes. Importantly, lack of meaning in work is a crucial documented driver of physician burnout. Clinical encounters perceived as meaningful by physicians could therefore potentially positively impact physician well-being. Here we reflect on the potential of interventions that aim to enhance the patient-physician interaction, such as shared decision making, to improve physician well-being by facilitating interactions with patients that are perceived as meaningful.

## Editorial

Physicians have higher rates of burnout, depressive symptoms, and suicide risk than the general population and the trend is getting worse, with 54% of American physicians affected by burnout in 2014 [1]. Physician burnout, a term used to describe emotional exhaustion, fragmentation, detachment, depersonalization, and a sense of reduced personal accomplishment in physicians, is caused by an individual’s stress or lack of resilience, isolation, and a loss of teamwork in an already stressed and potentially toxic working environment. It is associated with depression, substance abuse, and an increased suicide risk. Physician burnout has also been shown to negatively impact quality and safety of patient care, patient satisfaction and workforce turnover [1]. While changes in the practice environment such as an increasing administrative workload and pressure to see more patients faster, reduce costs, and improve quality and outcomes can contribute to burnout, suboptimal features of the doctor-patient relationship can also be a source of distress for physicians, particularly in the context of these other demands. Patients’ challenging health concerns and physicians’ difficulties in satisfactorily addressing patients’ needs, can strain well-being for both patients and physicians. Mindful engagement with patients, while often beneficial to physicians, is limited by efficiency pressures and clerical burdens. As physicians face these demands, they experience physiological changes, often referred to as allostatic load, with ill consequences to physicians’ well-being [2]. Allostatic overload is associated with a fluctuating or heightened neural or neuroendocrine response, which is a risk factor for sickness, including chronic depression and anxiety, lack of empathy and defensive emotional withdrawal (burnout) [2].

However, just as the clinical encounter, defined as a direct interaction between patient and health professional during which an assessment or clinical activity is performed, can contribute to burnout, it is sensible to postulate that it can contain remedies for burnout and promote well-being. That is, favorable sociophysiological engagements could contain the potential to positively influence the health of both parties, patient and physician. Caring within the patient-physician interaction is a central element of the clinical encounter. Social and emotional support has been linked to improved health outcomes in patients [3]. Patients who feel heard, understood, valued, respected and cared may be more likely to feel satisfied and grateful for their medical care. These positive feelings may have positive effects on physicians. Some studies suggest that supporting could be more beneficial on health and well-being than being supported [3]. The satisfaction that comes from helping somebody with whom we empathize and who appreciates our support in a caring clinical encounter may well help to reduce burnout [2]. How then can we influence whether our sociophysiological engagement during the clinical encounter will shift us towards sickness and burnout or health and well-being? Are there identifiable characteristics of clinical encounters that are mutually beneficial for patients and physicians? And if so, how do we make favorable encounters more prevalent?

Among documented drivers of physician burnout, lack of meaning in work appears crucial [1]. It follows that clinical encounters perceived as meaningful by physicians could potentially positively impact physician well-being. An American study in which physicians were asked to write about a meaningful work-related experience identified three major themes: 1) having been part of a profound event or emotional experience with a patient or sharing or reflecting on their own life experiences, which resulted in a fundamental change in physicians’ perspective about themselves, human nature, illness, and patient care; 2) connecting with patients in moments of intimacy, e.g. when physicians were moved by their patients’ humanity; and 3) making a difference in someone’s life, often the physicians themselves being the principal therapeutic agents, e.g. when physicians’ mere presence was healing and comforting to patients [4]. Shared decision making, the work that patients and physicians do together in conversation to arrive at the best possible approach to address the patient’s situation, is likely to promote these meaningful work experiences. By improving the most meaningful activity for most physicians, their engagement with patients, shared decision making could reduce physician burnout and promote well-being.

Our anecdotal experience from observing video recordings of clinical encounters in trials of shared decision-making tools is that collaborative communication and deliberation is associated with expressions of positive emotion in patients and physicians. Unfortunately, to our knowledge, studies of the impact of shared decision making do not routinely assess the effect this practice can have on physicians, although those that assessed physician experiences showed promising results [5].

Physician burnout (characterized by emotional and physical exhaustion, feelings of depersonalization, detachment and cynicism, and a reduced sense of competence or achievement in one’s work) and well-being are personal traits that do not tend to change immediately. Thus, to elucidate the potential role of shared decision making to improve physician well-being, long term implementation studies are needed. These studies should determine the extent to which physicians experience patient encounters as ‘meaningful,’ that is, professionally satisfying, fulfilling, in line with why they chose to become a physician.

By highlighting the potential of caring clinical encounters to positively contribute to patients’ and physicians’ well-being we do not wish to diminish the importance of addressing organizational factors that contribute to physician burnout, nor do we wish to promote shared decision making primarily as a potential tactic to improve physician well-being. Shared decision making is justified purely on deontological grounds. Physicians should be empathic, supportive and provide a safe environment to patients. Empathy, which leads to engaged and effective patient-physician interaction, offers a middle ground between physicians being emotionally over-involved, which could result in emotional distress, and physicians being over-detached, which can have detrimental consequences for physicians and patients. Beyond deontological reasons, a positive effect on physician well-being could add to the long list of reasons to invest in achieving better patient-physician interactions.

In summary, meaningful work prevents burnout among physicians, and positive clinical encounters with patients are correlated with physicians perceiving work as meaningful. We thus propose that interventions to enhance the patient-physician interaction, such as shared decision making, could potentially positively impact on physician burnout and well-being. Long term implementation studies are needed to assess the impact of shared decision making on physician burnout.
